# Failure to use health services by people with Chagas disease: Multilevel analysis of endemic area in Brazil

**DOI:** 10.1371/journal.pntd.0010785

**Published:** 2022-09-19

**Authors:** Renata Fiúza Damasceno, Ester Cerdeira Sabino, Antonio Luiz Pinho Ribeiro, Ariela Mota Ferreira, Léa Campos de Oliveira-da Silva, Cláudia Di Lorenzo Oliveira, Clareci Silva Cardoso, Thallyta Maria Vieira, Desirée Sant’ Ana Haikal

**Affiliations:** 1 Graduate Program in Health Sciences, State University of Montes Claros, Montes Claros, Minas Gerais, Brazil; 2 Institute of Tropical Medicine, University of São Paulo, São Paulo, Brazil; 3 Department of Internal Medicine, Federal University of Minas Gerais, Belo Horizonte, Minas Gerais, Brazil; 4 Federal University of São João del-Rey, Research Group in Epidemiology and New Technologies in Health–Centro Oeste Campus, Divinópolis, Minas Gerais, Brazil; Federal University of Ceará, Fortaleza, Brazil, BRAZIL

## Abstract

This study aimed to assess the prevalence of non-use of health services in the last year by people with Chagas disease (CD) in an endemic area in Brazil and the contextual and individual factors associated with this non-use. This is a multilevel study that considered contextual and individual data. Contextual data were collected from official publicly accessible databases of the Brazilian government, at the municipal level. The individual data came from the first follow-up of a Brazilian cohort that assessed patients with CD in 21 municipalities in endemic area for the disease. The sample consisted of 1,160 individuals with CD. The dependent variable “use of health services in the last year” was categorized as yes vs. no. The analysis was performed using Poisson regression with robust variance. The prevalence of non-use of health services in the last year was 23.5% (IC95%: 21.1–25.9). The contextual factor “larger population” (PR: 1.6; 95% CI = 1.2–2.0) and individual factors related to the lower severity of the disease as a functional class without limitations (PR: 1.6; 95% CI = 1.2–2.1) and unaltered N-terminal pro b-type natriuretic peptide levels (PR: 2.2; 95% CI = 1.3–3.6) increased the prevalence of non-use of the health service in the last year by people with CD. The results of this study showed that individual determinants are not isolated protagonists of the non-use of health services in the last year by people with CD, which reinforces the need for public policies that consider the contextual determinants of the use of health services by populations affected by the disease.

## Author summary

Chagas disease (CD) is a parasitic infection caused by *Trypanosoma cruzi* (*T*. *cruzi*) that predominantly affects poor and vulnerable populations. It is estimated that around six million people are infected with *T*. *cruzi*, most of them in endemic areas of Latin American countries. It is recommended that individuals with CD be followed up by health services through one to two medical consultations per year. Identifying groups of people with CD who are more vulnerable in relation to their health status, whether due to the unavailability of health services or the types of behavior that lead to the non-use of available services, can contribute to reducing morbidity and mortality from the disease. Our study evaluated the prevalence of non-use of health services in the last year by 1,160 people with CD in an endemic area in Brazil and the contextual and individual factors associated with this non-use. The study results showed that almost a quarter of these patients had not had a medical appointment in the last year. “Living in cities with a larger population” and “having a lower severity of the disease” influenced the non-use of the health service in the last year by these patients. These results reinforce the need for public policies that consider the contextual determinants of the use of health services by populations affected by the disease.

## Introduction

Chagas disease (CD) is a systemic and chronic parasitic infection caused by *Trypanosoma cruzi* (*T*. *cruzi*) that predominantly affects poor and vulnerable populations. With globalization and constant migratory flows, CD is present on almost every continent. It is estimated that in the world more than 7,500 deaths from CD occur per year, and that approximately six million people are infected with *T*. *cruzi*, most of them in endemic areas of Latin American countries [1,2,3]. In the Americas, CD is the parasitic disease with the highest mortality burden and disability-adjusted life years (DALYs) [3]. In Brazil it is estimated that the prevalence of CD is around 0.6%, which corresponds to more than 1.1 million infected individuals [4]. This scenario points to a major challenge for the country’s health system: ensuring access to comprehensive care for over a million people with CD [5].

Although annual rates of incidence and prevalence of CD have fallen as a result of measures to control the disease and improve quality of life, care for patients with CD is still a major challenge for health systems in endemic [6,7] and non-endemic countries [8,9]. It is recommended that individuals with CD be followed-up by health services through one to two medical consultations per year for the early detection of the clinical progression of the disease, treatment, and promotion of healthy lifestyle habits [10,11,12]. It is noteworthy that medical assistance to patients with CD is influenced by the economic and social context of the regions where patients live [13].

In Brazil, individuals with CD should be monitored longitudinally in public Primary Health Care (PHC) services through periodic medical consultations and, when necessary, referred to specialized health services [12]. The use of health services in the country is provided in three ways: public, private, and supplementary, with the public sector being the main provider [14,15]. It is estimated that more than 70% of the Brazilian population depends on the Sistema Único de Saúde (SUS) for access to health care [16].

The use of health services is an important marker for assessing access and equity in health systems [17]. In Brazil, people with chronic non-communicable diseases (NCDs) use health services more than people without NCDs [18]. To date, studies on the non-use of health services by people with CD have not been identified in the national and international literature.

The assumed universality of access to the health system and the well-known social inequality in Brazil offer interesting opportunities for analyzing the contextual and individual determinants of the use of health services [19] by populations with neglected diseases in the country. In this context, identifying groups of people with CD who are most vulnerable regarding their health status, whether due to the unavailability of health services or the types of behavior that lead to the non-use of available services [17], may contribute to the development and implementation of effective public policies for the comprehensive care of populations with CD. Thus, the aim of this study was to assess the prevalence of non-use of health services in the last year by people with CD in an endemic area in Brazil and the contextual and individual factors associated with this non-use.

## Methods

### Ethics statement

This study was approved by the National Research Ethics Commission (CONEP: 179,685/2012). All subjects agreed to participate and signed the informed consent form prior to the beginning of the study.

### Study design

This is a multilevel study organized based on the Behavioral Model of the Use of Health Services by Andersen & Davidson [17] that considered contextual and individual data. Contextual data refer to the municipal level and were collected from official publicly accessible databases of the Brazilian government. The individual data were collected during the first follow-up of a prospective cohort study with patients with CD called SaMi-Trop (Center for Research on Biomarkers in Neglected Tropical Diseases in São Paulo / Minas Gerais).

The SaMi-Trop cohort is a multicenter study resulting from the cooperation between four Brazilian public universities: University of São Paulo (USP), Federal University of Minas Gerais (UFMG), Federal University of São João del-Rey (UFSJ) and State University of Montes Claros (Unimontes). The SaMi-Trop methodology has been presented in detail in previous publications [20,21].

### Research environment

The SaMi-Trop cohort covers an endemic area for CD in the state of Minas Gerais, Brazil. This area consists of 21 municipalities which are located in two remote regions of the state: the northern region of the state and the Jequitinhonha Valley region. Most of these municipalities were small in size, with a high percentage of rural population and high social vulnerability—with the absence or insufficiency of some assets related to urban infrastructure, human capital and income and work, which should, in principle, be available to every citizen, by virtue of State action [22]. In relation to the health sector, most municipalities had 100% coverage of the Family Health Strategy (FHS) which did not offer specialized services [23]. The FHS is the main PHC model in the country and provides health care for the population of a defined territory. The services are provided by a team that includes a doctor, a nurse, a nursing technician or assistant, and community health workers [24]. In Brazil, PHC is the gateway to public health care, being offered in all municipalities. The specialized services in contrast, are regionalized and organized into municipalities with a larger population and better health structure [25].

### Study population and recruitment

Patients over 18 years of age who reported having CD, while undergoing electrocardiogram (ECG) exam by a Telehealth program in 2012, were recruited to participate in the cohort. The Telehealth Network of the state of Minas Gerais offers remote support to public PHC services through ECG reports and teleconsulting in several areas of health [26]. The individuals included in the cohort participated in two assessments to date, with an interval of two years between them.

**Fig 1:**
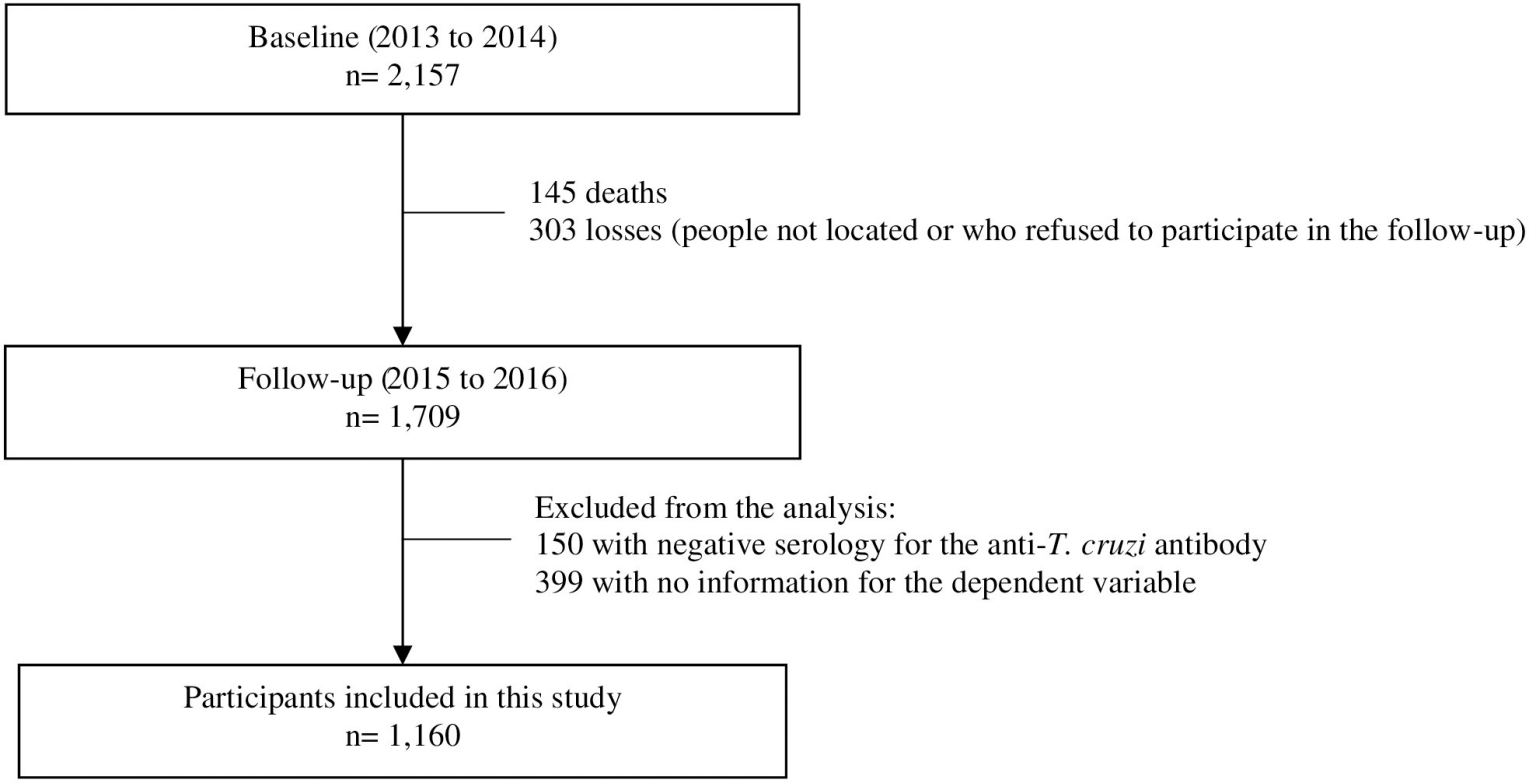
Flowchart of included, lost and excluded CD patients of the study. SaMi-Trop Project, Minas Gerais, Brazil.

At baseline, which took place from 2013 to 2014, 2,157 individuals participated. In the first follow-up, which took place from 2015 to 2016, 1,709 individuals were assessed. Between stage one and stage two there were 303 losses (people not located or who refused to participate in the follow-up) and 145 deaths. A total of 1,709 individuals were considered eligible to participate in this study, of which 150 were excluded from the analysis because they presented negative serology for the anti-*T*. *cruzi* antibody, and 399 for not presenting an answer for the dependent variable. Thus, 1,160 individuals with CD were included in this study (Fig 1).

### Variables and data sources

Based on the theoretical model adopted in this study [17], the use of health services in the last year was adopted as an outcome of interest in this study. This variable was built from the question “How long has it been since your last medical consultation related to CD?”, and later categorized into “use of health services in the last year” (yes vs. no).

The choice of independent variables (contextual and individual) was also based on the Behavioral Model of the Use of Health Services [17]. According to this model, the use of health services is influenced at the contextual and individual levels, by the following components: predisposing characteristics (existing conditions that predispose individuals to use health services or not, even if these conditions are not directly responsible for use); enabling factors (conditions that facilitate or hinder the use of health services); and need (conditions recognized by individuals or health service providers as health care demanders). These components modulate health behavior (personal health practices that influence an individual’s health status) and an individual’s health outcomes (perceived health status of an individual; health status assessed by a health professional; and satisfaction of the individual in relation to the health care they receive).

The contextual data of the 21 municipalities participating in SaMi-Trop came from official publicly accessible databases of the Brazilian government: Atlas of Human Development in Brazil (Atlas do Desenvolvimento Humano no Brasil—AtlasBr) [22]; Ministry of Health (Ministério da Saúde—MS) [27] and Brazilian Institute of Geography and Statistics—Cities (Instituto Brasileiro de Geografia e Estatística—Cidades—IBGE Cidades) [28]. Data were collected on six variables related to sociodemographic aspects, public health policies, and health indicators. The contextual variables, with the exception of the Municipal Human Development Index (MHDI) variable, are numeric and were dichotomized considering the 25th percentile when it was a negative measure (low values indicated the best situation) or the 75th percentile when it was a positive measure (high values indicated the best situation). The objective of this dichotomization was to separate 25% of the better-off municipalities vs. 75% of the municipalities in the worst situation, since the majority of the municipalities included in this study had low socioeconomic status.

The individual data were collected during the first follow-up of the cohort through interviews and peripheral blood tests. At this stage, patients with CD participating in the cohort were invited to attend a primary care unit in the municipalities on the day and time scheduled for an interview and clinical evaluation, which were carried out by researchers from SaMi-Trop. The interview included socio-demographic data, data on the clinical and therapeutic history of CD, and data on lifestyle, quality of life, and the use of health services (Table 1).

**Table 1:** Characterization of the study variables based on the levels and components of the Behavioral Models of the Use of Health Services by Andersen & Davidson.

Levels	Components	Variable	Description	Data source and year
**Level 1** Contextual characteristics	Predisposing characteristics	Population	Indicates the number of people residing in the municipality (up to 31 thousand people; >31 thousand people).	AtlasBr, 2010 [22]
		MHDI	Measure composed of indicators of three dimensions of human development: longevity, education, and income. Categorized according to the index’s own recommendation, and later, dichotomized (high and medium *vs*. low).	AtlasBr, 2010 [22]
		GINI Index	Index that measures the degree of concentration of income in a given group (>0.464; up to 0.464).	AtlasBr, 2010 [22]
	Enabling Factors	Health insurance coverage	Percentage of health plan beneficiaries in relation to the population in a specific area (up to 3%; >3%).	MS, 2017 [27]
		FHS coverage	Percentage of the population served by Family Health Strategy teams (<100%; 100%).	MS, 2017 [27]
	Need	Child mortality	Infant mortality rate per 1,000 live births (>8.13; up to 8.13).	IBGE Cidades, 2014 [28]
**Level 2** Individual Characteristics	Predisposing characteristics	Gender	Female; Male.	1° follow-up Cohort SaMi-Trop, 2015/2016
		Age	Over 60; Up to 60 years.	
		Color	Self-declared skin color (not white; white).	
		Marital status	Without stable union; Stable union.	
		Literacy	No; Yes.	
	Enabling Factors	Income	Up to 1 minimum wage; Above 1 minimum wage (dichotomized considering the value of the minimum wage in force in the country at the time of data collection—R$724.00 per month).	
		Distance from PHC service	Distance from the individual’s residence to the nearest PHC service (more than 100 km; 6 to 99 km; 0 to 5 km).	
	Need	Diabetes mellitus	Self-report of diabetes diagnosis mellitus (yes; no).	
		Hypertension	Self-report of diagnosis of systemic arterial hypertension (yes; no).	
		Functional class	Stratifies the degree of limitation imposed by heart failure for the individual’s daily activities [29] (with limitations—classes II, III and IV; without limitations—class I).	
		N-terminal pro b-type natriuretic peptide (NT-proBNP)	NT-proBNP levels are accurate discriminators of the diagnosis of heart failure, powerful predictors of death, and assist in the risk stratification of patients with CD [30,31] (altered; not altered).	
		Time since diagnosis of CD	Self-report of the time since diagnosis of CD (more than 10 years; 1 to 10 years; less than 1 year).	
		Self-perceived health	Negative; Positive.	
**Level 3** Health Behaviors	Personal health practices	Physical activity	No; Yes.	1^st^ follow-up SaMi-Trop cohort, 2015/2016
		Alcohol use	Frequent use of alcohol (consumed 3 to 5 times a week/consumed every day); Infrequent alcohol use (did not consume/consumed less than once a week/consumed 1 to 2 times a week).	
		Smoking	Smoker (smoked at the time of data collection); Non-smoker (ex-smokers and those who had never smoked)	

### Statistical analysis

A descriptive analysis of the individual variables was subsequently performed, the absolute (n) and relative (%) frequencies were estimated, and the bivariate analysis was performed using Pearson’s chi-square test. Individual variables with a value of p ≤0.20 were selected to initially compose the multivariate model, respecting the entry by levels.

After confirming the absence of multicollinearity between contextual and individual variables (correlation <0.7), Poison Regression analysis with Robust Variance was performed. For the multilevel analysis, the model of fixed effect and random intercept (mixed) was used [32]. A priori, an empty model was considered (only with random intercept and the dependent variable, without the other variables). Subsequently, variables from the first level (contextual variables), followed by variables from the second (individual variables related to individual characteristics) and third level (individual variables related to health behaviors) were included, as proposed by the theoretical model adopted in this study (Table 1). The Prevalence Ratio (PR) and 95% CI were calculated to quantify associations between the outcome and the contextual and individual variables. The model was adjusted at the introduction of each level, keeping in the final model only those with statistical significance (p ≤0.05). Deviance, represented by “-2 loglikelihood”, was used to assess the quality of the fit.

Descriptive and bivariate analyzes were performed using SPSS statistical software version 18.0 (IBM SPSS, IBM Corp., Armonk, United States) and multivariate analysis with STATA version 14.0 (StatCorp, College Station, Texas, 270).

## Results

A total of 1,160 people with CD from 21 municipalities in endemic area for disease in the state of Minas Gerais, Brazil participated in this study. Among the participants, 67.8% were female, 58.4% were aged up to 60 years, 60% were literate, 76.8% reported having been diagnosed with CD for more than 10 years, and 77.6% had positive self-perceived health (Table 2).

**Table 2:** Descriptive and bivariate analysis of individual characteristics and health behaviors of people with Chagas disease and its association with not using health services in the last year. Minas Gerais, Brazil (n = 1,160).

Variables	Descriptive	Bivariate		p-value ^π^
		Use of health services in the last year		
*Individual characteristics*	n (%)	Yes (%)	No (%)	
**Predisposing characteristics**				
Gender				0.081^¥^
Female	787 (67.8)	590 (75.0)	197 (25.0)	
Male	373 (32.2)	297 (79.6)	76 (20.4)	
Age				0.004^¥^
60 years or older	482 (41.6)	389 (80.7)	93 (19.3)	
Up to 60 years	678 (58.4)	498 (73.5)	180 (26.5)	
Self-reported skin color*				0.034^¥^
Non-white	897 (77.7)	673 (75.0)	224 (25.0)	
White	258 (22.3)	210 (81.4)	48 (18.6)	
Marital status*				0.453
Single, widowed or divorced	399 (34.5)	310 (77.7)	89 (22.3)	
Married or cohabiting	758 (65.5)	574 (75.7)	184 (24.3)	
Literacy*				0.010^¥^
No	462 (40.0)	371 (80.3)	91 (19.7)	
Yes	694 (60.0)	512 (73.8)	182 (26.2)	
**Enabling factors**				
Family income*				0.094^¥^
Up to minimum wage	627 (54.2)	467 (74.5)	160 (25.5)	
Above minimum wage	530 (45.8)	417 (78.7)	113 (21.3)	
Distance from the Health Unit*				0.870
Over 100 km	54 (5.5)	43 (79.6)	11 (20.4)	
6 to 99 km	262 (26.6)	200 (76.3)	62 (23.7)	
0 to 5 km	668 (67.9)	512 (76.6)	156 (23.4)	
**Need**				
Functional class				< 0.001^¥^
With limitations	471 (41.0)	390 (82.2)	81 (17.2)	
Without limitations	679 (59.0)	488 (71.9)	191 (28.1)	
NT-proBNP				< 0.001^¥^
Altered	148 (12.8)	132 (89.2)	16 (10.8)	
Normal	965 (83.2)	723 (74.9)	242 (25.1)	
Diabetes mellitus				0.603
Yes	129 (11.1)	101 (78.3)	28 (21.7)	
No	1031 (88.9)	786 (76.2)	245 (23.8)	
Systemic arterial hypertension*				0.016^¥^
Yes	771 (66.5)	606 (78.6)	165 (21.4)	
No	389 (33.5)	281 (72.2)	108 (27.8)	
Time since CD diagnosis*				0.409
More than 10 years	696 (76.8)	548 (78.7)	148 (21.3)	
1 to 10 years	199 (22.0)	151 (75.9)	48 (24.1)	
Less than 1 year	11 (1.2)	10 (90.9)	1 (9.1)	
Self-perceived health*				0.003^¥^
Negative	258 (22.4)	215 (83.3)	43 (16.7)	
Positive	895 (77.6)	666 (74.4)	229 (25.6)	
***Health behaviors***				
**Personal health practices**				
Physical activity*				0.046^¥^
No	279 (24.1)	201 (72.0)	78 (28.0)	
Yes	881 (75.9)	686 (77.9)	195 (22.1)	
Alcohol use*				0.051^¥^
Frequent alcohol use	22 (1.9)	13 (59.1)	9 (40.9)	
Infrequent alcohol use	1137 (98.1)	874 (76.9)	263 (23.1)	
Smoking *				0.842
Smoker	61 (5.3)	46 (75.4)	15 (24.6)	
Non-smoker	1099 (94.7)	841 (76.5)	258 (23.5)	

*Variation of the n = 1.160 because of missing information;

^*π*^ Pearson’s chi-squared test;

^¥^ p≤ 0.20

The prevalence of non-use of health services in the last year related to CD in this population was 23.5% (IC95%: 21.1–25.9), varying from 0% to 37.5% in different municipalities. In the bivariate analysis (Table 2), the individual variables referring to the “individual characteristics” and “health behaviors” levels selected to compose the multiple model (p ≤0.20) were: gender, age, self-declared skin color, literacy, income, functional class, NT-proBNP, severe arterial hypertension, self-perceived health, physical activity, and alcohol use.

According to the adjusted multiple model (Table 3), only one contextual variable influenced the outcome. The prevalence of non-use of health services in the last year was higher among patients with CD living in municipalities with a larger population. At level 2 (individual characteristics), two individual variables remained in the model after adjustment: functional class and NT-proBNP. This result revealed a higher prevalence of non-use of health services in the last year among patients without functional class limitations and without alterations in NT-proBNP levels. No individual variable referring to level 3 (health behaviors) remained in the model after adjustment.

**Table 3:** Final model of the Multilevel Poisson Regression of the factors associated with the non-use of health services in the last year by people with Chagas disease. Minas Gerais, Brazil (n = 1,160).

Models	Variables	PR (IC95%)	p-value
**Level 1 *Contextual Characteristics***	Population		
	Smaller population	1	
	Larger population	1.6 (1.1–2.2)	0.008
Deviance (-2log Log likelihood) = **131.860.510**			
**Level 2** *Contextual Characteristics* ***Individual Characteristics***	Population		
	Smaller population	1	
	Larger population	1.6 (1.2–2.0)	< 0.001
	Functional class		
	With limitations	1	
	Without limitations	1.6 (1.2–2.1)	0.001
	NT-proBNP		
	Altered	1	
	Not altered	2.2 (1.3–3.6)	0.003
Deviance (-2log Log likelihood) = **122.272.774**			

## Discussion

The results of this study showed that almost a quarter of patients with CD residing in cities in the endemic area in Brazil did not have a medical consultation in the last 12 months related to the disease. The contextual factor "population" (larger population) and individual factors related to lower disease severity (functional class without limitations and non-substituted NT-proBNP levels) were associated with a higher prevalence of non-use of the health service in the last year by these patients.

The use of the health service in the last year is recognized as an important marker of access to health care [17]. The European Union has defined the one-year recall period for assessing the use of health services as a way of ensuring comparability between member countries [33]. In Brazil, surveys with national coverage also use the one-year recall period to assess the use of health services in the country [34].

The prevalence of non-use of health services in the last year among patients with CD was similar to the prevalence found for the general Brazilian population (23.8%) and slightly higher than the prevalence found for the general population of the state of Minas Gerais (21.7%) [16]. No previous studies were identified in the national and international literature that pointed out the prevalence of non-use of health services by people with CD or other infectious and parasitic diseases, which made comparisons of this nature impossible.

In Brazil, people with chronic non-communicable diseases (NCDs) used health services twice as often compared to people without NCDs [18]. In this study, most patients with CD who used health services in the last year had overlapping other diseases, such as systemic arterial hypertension (66.5%) and diabetes mellitus (11.1%). Thus, considering the recommendation that people with CD be followed-up by health services through at least one medical consultation per year [11,12], the prevalence of non-use of health services in the last year among CD patients was high.

In the theoretical model adopted in this study [17], the use of health services is influenced by the contextual and individual levels. At the contextual level, the findings of this study pointed out that only the population size of the patient’s municipality of residence affected the non-use of health services in the last year by populations with CD. Living in municipalities with a larger population was consistently associated with a higher prevalence of non-use of health services in the last year by the participants in this study.

Regardless of the FHS coverage, what seems to have favored the non-use of health services by patients with CD in the municipalities with a larger population was the difficulty of access to the health services offered. In Brazil, access to health services through SUS is a social right. The PHC services, which are responsible for offering periodic medical consultations to patients with CD, are public [35]. However, determinants inherent to the structure and organization of health services can generate barriers to access care for patients with CD in different regions of the country [36].

In Brazil, around 23% of the municipalities are medium (25 to 100 thousand inhabitants) and large (more than 100 thousand inhabitants) [37]. Municipalities with a larger population have more complex health systems and greater difficulty in structuring PHC and consolidating a preventive health care model [38]. In larger municipalities, it is also observed that the availability of health services may not be known to everyone, and the degree of information about the services available varies between different population groups [39]. Reports by PHC doctors in a medium-sized municipality endemic for CD pointed out that the disease was a hidden problem for PHC. According to the reports of these doctors, the lack of registration of patients with CD in PHC services was one of the main factors that contributed to the invisibility of the disease in the health system [36].

In smaller municipalities, residents and health workers are more likely to get to know each other and have a closer relationship, which facilitates access to information and the scheduling of consultations and exams [40]. A study that evaluated access to PHC services in small municipalities in Brazil showed that the functioning of these services on all days of the week, opening hours appropriate to the needs of patients, and ease in scheduling appointments were factors that enabled the use of health services in these municipalities, promoting more equitable access [41].

Regarding the individual level, the non-use of health services in the last year by patients with CD was influenced exclusively by health needs. Presenting functional class without limitations and levels of NT-proBNP without alterations were associated with a higher prevalence of non-use of health services in the last year by these patients. This finding shows that the use of services in the last year by patients with CD was motivated by the presence of symptoms and severity of the disease. A study carried out with PHC doctors from an endemic region in Brazil also pointed out that the search for medical care by patients with CD also happened only when there was the presence of some complication [36].

The limitation of the functional class is known to be associated with death caused by increased myocardial dysfunction. NT-proBNP levels are accurate discriminators of the diagnosis of heart failure, powerful predictors of death, and assist in the risk stratification of patients with CD [31]. Patients with symptomatic or asymptomatic CD should be followed-up by the health services through annual medical consultations for early detection of the clinical progression of the disease and early implementation of therapy for the treatment of visceral complications. Regular follow-up of these patients in health services can contribute to a reduction in mortality from CD [10] in both endemic and non-endemic regions.

In Brazil, one of the main reasons for seeking care in health services is illness or disease treatment (48.2%) [16], which reveals clinical diagnosis as a condition for frequent use of health services [42]. According to a systematic review of qualitative studies, factors such as the absence of symptoms and the impact on daily activities have negatively influenced the search for medical care by patients with CD in endemic areas [43].

The failure to use health services in the last year by patients with CD motivated by the absence of symptoms and the severity of the disease can also be an indicator of the lack of public health policies for comprehensive care to populations affected by the disease. Failure to perform screening and early diagnosis of the disease may contribute to the patient’s lack of perception of the need to use health services to prevent complications. Thus, there is an urgent need to develop approaches for comprehensive, sustained, efficient, timely, adequate, and accessible medical care, focused on universal coverage and comprehensive care for populations with CD [44].

Individual factors such as gender, age, self-declared skin color, education, and income did not influence the non-use of health services in the last year by patients with CD participating in this study, possibly because this population was relatively homogeneous in terms of sociodemographic profile.

This study has some limitations. There is a certain homogeneity of the population in terms of sociodemographic characteristics. The variable “use of health services in the last year” was analyzed retrospectively, based on the respondent’s memory. And, despite the short period of interest (12 months), the presence of memory bias cannot be ruled out. Contextual data refer to time periods different from individual data due to the unavailability of updated data in the official publicly accessible databases of the Brazilian government at the time of collection. The participation of CD patients in a cohort study may motivate them to seek medical care. However, this is the first study with a multilevel approach that assessed the contextual and individual influence on the non-use of health services by populations affected by CD. The results of this study have important implications for the planning of health care policies aimed at these populations.

This study revealed that the non-use of health services in the last year by patients with CD from endemic area in Brazil was influenced by the population size of the municipality where these patients live and by the absence of symptoms and severity of the disease. According to these results, individual determinants are not isolated protagonists of the non-use of health services by people with CD. The context in which these people live also determines the non-use of health services, which reinforces the need for public policies that consider the contextual determinants of the use of health services by populations affected by the disease.

## Supporting information

S1 STROBE Checklist Checklist. (DOCX)

S1 Database Data that underlies this paper. (XLSX)
